# In Vitro Evaluation and In Silico Calculations of the Antioxidant and Anti-Inflammatory Properties of Secondary Metabolites from *Leonurus sibiricus* L. Root Extracts

**DOI:** 10.3390/molecules28186550

**Published:** 2023-09-10

**Authors:** Anna Merecz-Sadowska, Przemysław Sitarek, Tomasz Kowalczyk, Marcin Palusiak, Marta Hoelm, Karolina Zajdel, Radosław Zajdel

**Affiliations:** 1 Department of Economic and Medical Informatics, University of Lodz, 90-214 Lodz, Poland; radoslaw.zajdel@uni.lodz.pl; 2 Department of Medical Biology, Medical University of Lodz, 90-151 Lodz, Poland; przemyslaw.sitarek@umed.lodz.pl; 3 Department of Molecular Biotechnology and Genetics, Faculty of Biology and Environmental Protection, University of Lodz, 90-237 Lodz, Poland; tomasz.kowalczyk@biol.uni.lodz.pl; 4 Theoretical and Structural Group, Department of Physical Chemistry, Faculty of Chemistry, University of Lodz, 90-236 Lodz, Poland; marcin.palusiak@chemia.uni.lodz.pl (M.P.); marta.hoelm@chemia.uni.lodz.pl (M.H.); 5 Department of Medical Informatics and Statistics, Medical University of Lodz, 90-645 Lodz, Poland; karolina.smigiel@umed.lodz.pl

**Keywords:** anti-inflammatory properties, antioxidant properties, AtPAP1, *Leonurus sibiricus* L., root extract, transgenic root extract

## Abstract

*Leonurus sibiricus* L. has great ethnobotanical and ethnomedicinal significance. This study aimed to assess the antioxidant and anti-inflammatory properties of *Leonurus sibiricus* L. transgenic roots extracts transformed by *Rhizobium rhizogenes*, with and without the AtPAP1 transcriptional factor. The study determined the total phenolic and flavonoid contents, as well as in vitro antioxidant assays, including hydrogen peroxide and nitric oxide scavenging activity. In addition, in silico computational studies and molecular docking were conducted to evaluate the antioxidant and anti-inflammatory potential of the identified compounds. The ligands were docked to NADPH oxidase, cyclooxygenase 2,5-lipoxygenase, inducible nitric synthase and xanthine oxidase: enzymes involved in the inflammatory process. The total phenolic and flavonoid contents ranged from 85.3 ± 0.35 to 57.4 ± 0.15 mg/g GAE/g and 25.6 ± 0.42 to 18.2 ± 0.44 mg/g QUE/g in hairy root extracts with and without AtPAP1, respectively. H_2_O_2_ scavenging activity (IC_50_) was found to be 29.3 µg/mL (with AtPAP1) and 37.5 µg/mL (without AtPAP1 transcriptional factor), and NO scavenging activity (IC_50_) was 48.0 µg/mL (with AtPAP1) and 68.8 µg/mL (without AtPAP1 transcriptional factor). *Leonurus sibiricus* L. transformed root extracts, both with and without AtPAP1, are a source of phytochemicals belonging to different classes of molecules, such as flavonoids (catechin and rutin), phenolic compounds (caffeic acid, coumaric acid, chlorogenic acid, ferulic acid) and phenylpropanoid (verbascoside). Among the radicals formed after H removal from the different -OH positions, the lowest bond dissociation enthalpy was observed for rutin (4′-OH). Rutin was found to bind with cyclooxygenase 2, inducible nitric synthases and xanthine oxidase, whereas chlorogenic acid demonstrated optimal binding with 5-lipoxygenase. Therefore, it appears that the *Leonurus sibiricus* L. transformed root extract, both with and without the AtPAP1 transcriptional factor, may serve as a potential source of active components with antioxidant and anti-inflammatory potential; however, the extract containing AtPAP1 demonstrates superior activities. These properties could be beneficial for human health.

## 1. Introduction

Honeyweed or Siberian motherwort (*Leonurus sibiricus* L.), a member of the Lamiaceae family, is a herbaceous plant found in several countries across Asia, Africa and America. It has been widely used as a culinary ingredient and herbal medicine. *L. sibiricus* L. has been found to be effective in treating several health conditions including diabetes, hypertension, myocardial ischemia, chronic rheumatism and bronchitis. The therapeutic properties of *L. sibiricus* L. can be attributed to the presence of secondary metabolites, which are important biological active molecules. Numerous phenolics, diterpenes and alkaloids have been identified in various extracts from the aerial parts, leaves and roots [1,2,3]. Studies have also found that the production of selected secondary metabolites can be enhanced by manipulating their synthetic pathways [4].

Phenolics, which are important antioxidants found in plants, can have molecular structures that vary from simple to complex. They are defined as molecules that have an aromatic ring with one or more hydroxyl groups, encompassing both low-molecular-weight compounds with a single aromatic ring and large polyphenolic molecules. The antioxidant activity of phenolics depends on their structural properties. Bors proposes three criteria to explain the antioxidant behavior of phenolic compounds. The first is the presence of a catechol group on the B-ring, which increases the stability of the resulting antioxidant radical. The second is the presence of a 2,3 double bond combined with a 4-oxo group on the C-ring, which facilitates electron delocalization. The third is the presence of OH groups at positions 3 and 5 in combination with a 4-oxo group, which enables electron delocalization via hydrogen bonds [5,6,7].

There are five mechanisms that describe antioxidant reactions: hydrogen atom transfer (HAT), single electron transfer (SET), single-electron transfer followed by proton transfer (SET-PT), sequential proton loss electron transfer (SPLET) and transition metal chelation (TMC). The HAT mechanism involves the direct reaction of a phenolic antioxidant with a free radical, resulting in neutralization of the radical and formation of a radical form of the phenolic antioxidant. In the SET mechanism, the phenolic antioxidant molecule reacts with the free radical to produce a cationic radical form of the phenolic antioxidant and an anionic form of the radical. In the SET-PT mechanism, the first step is the same as SET, but in the second step, the cationic radical form of the phenolic antioxidant decomposes into a phenolic radical and a proton. The SPLET mechanism also involves a two-step reaction: the phenolic antioxidant first dissociates into an anionic form and a proton, and then these ions react with the free radical, generating a neutral molecule and a radical form of the phenolic antioxidant. In the TMC mechanism, each molecule that may dissociate has the ability to chelate heavy metals. Anions of polyphenols have significant abilities to chelate heavy metals [8,9,10]. 

While some reactive species are necessary for physiological functions, a large amount of these species may be harmful. Reactive oxygen species (ROS) have been proposed to impact the NF-κB signaling pathway, which is of central importance in inflammation. Inflammation is a complex response to internal and external stimuli that involves the infiltration of various types of inflammatory cells, such as neutrophils, monocytes and lymphocytes, to the site of the stimulus. Inflammatory cells at the site of inflammation release numerous inflammatory mediators including growth factors, chemokines, cytokines and reactive species that can cause tissue damage [11,12,13].

The canonical NF-κB pathway is primarily activated through the stimulation of proinflammatory receptors, including the tumor necrosis factor receptor (TNFR) family, Toll-Like receptor (TLR) family and cytokine receptors for the interleukins. Due to its role in inflammation, certain enzymes involved in ROS production are regulated as targets of NF-κB. These enzymes include NADPH oxidase (NOX), xanthine oxidase (XO), inducible nitric oxide synthase (iNOS), cyclooxygenase-2 (COX-2) and arachidonate 5-lipoxygenase (LOX-5). NADPH oxidase enzymes have total six subunits (Nox2/gp91phox, p22phox, p40phox, p47phox, p67phox, GTP-Rac) and are specifically responsible for ROS production. XO catalyzes the interconversion of xanthine and urate, but it exhibits low specificity, leading to the generation of superoxide and hydrogen peroxide when electrons are transferred to O_2_ instead of NAD^+^. iNOS primarily produces reactive nitrogen species (RNS) in the form of nitric oxide (NO), which can react with superoxide to form highly reactive peroxynitrite. COX-2 and LOX-5 contribute to increased levels of inflammatory prostaglandins H2 and leukotriene B4 [14,15,16,17].

Our objectives were to determine the total phenolic and flavonoid content of transgenic roots extracts transformed by *Rhizobium rhizogenes*, with and without the AtPAP1 transcriptional factor and evaluate their antioxidant and anti-inflammatory activities. Therefore, the study examines the potential of *L. sibiricus* L. root extracts to scavenge hydrogen peroxide and nitric oxide in vitro. In addition, in quantum mechanical prediction and molecular docking analysis were performed in silico to determine the antioxidant and anti-inflammatory potentials of the potential bioactive compounds.

## 2. Results

### 2.1. HPLC and LC-MS/MS Analyses of TR and AtPAP1 L. sibiricus L. Root Extracts

The quantitative determination of phytochemicals in the transformed *L. sibiricus* L. root extracts with (AtPAP1) and without (TR) AtPAP1 transcription factor was performed as described previously [18,19,20]. Briefly, HPLC analysis indicated that all compounds were present in greater amounts in the AtPAP1 root extract than the TR without construct. The dominant substance in both extracts was chlorogenic acid: its content in the AtPAP1 root extract was 4.7 times higher than in the TR root extract. The second most prevalent was caffeic acid, whose content was 2.7-times higher in the AtPAP1 root extract than in the TR root extract. The results are shown in Table 1.

**Figure 1:**
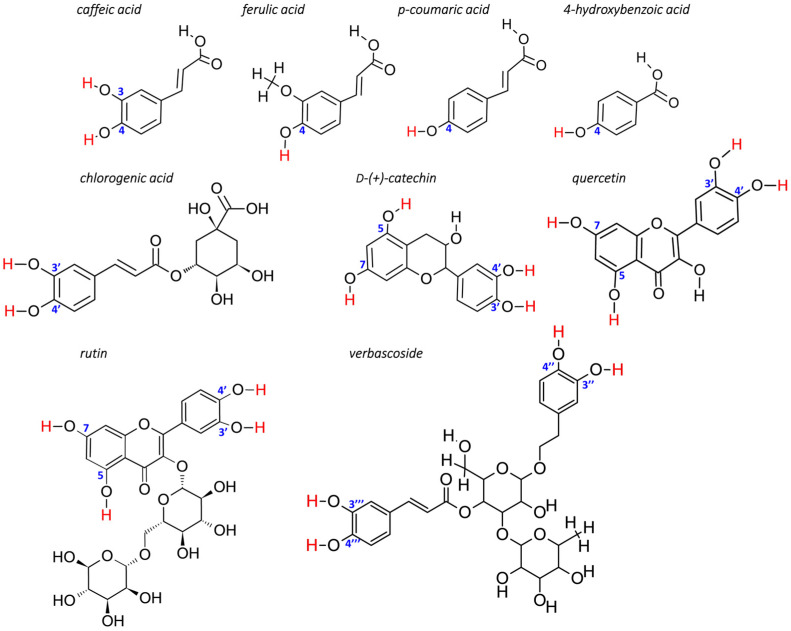
Schematic representation of molecules identified in TR and AtPAP1 *L. sibiricus* L. root extracts. The hydrogen atoms removed to form radicals are highlighted in red.

Schematic representation of molecules is shown in Figure 1. Phytochemicals belonged to different classes of molecules, such as phenolic compounds (caffeic acid, coumaric acid, chlorogenic acid, ferulic acid), flavonoids (catechin and rutin) and phenylpropanoid (verbascoside).

### 2.2. Total Phenolic and Flavonoids Content of the TR and AtPAP1 L. sibiricus L. Root Extracts

Total phenolic and flavonoid content were assessed employing the Folin–Ciocalteu and aluminum chloride colorimetric assays, respectively. The phenolic and flavonoid compounds present in the TR and AtPAP1 *L. sibiricus* L. root extracts were quantified in terms of gallic acid equivalents (GAEs) and quercetin equivalents (QUEs) per gram of dry extract weight. Among the extracts, the AtPAP1 root extract exhibited the highest total phenolic and flavonoid content. Specifically, the AtPAP1 root extract demonstrated 1.4 times greater total phenolic content and 1.3 times higher total flavonoid content compared to the TR root extract. Detailed results are presented in Table 2.

### 2.3. Hydrogen Peroxide Scavenging Activity

**Figure 2:**
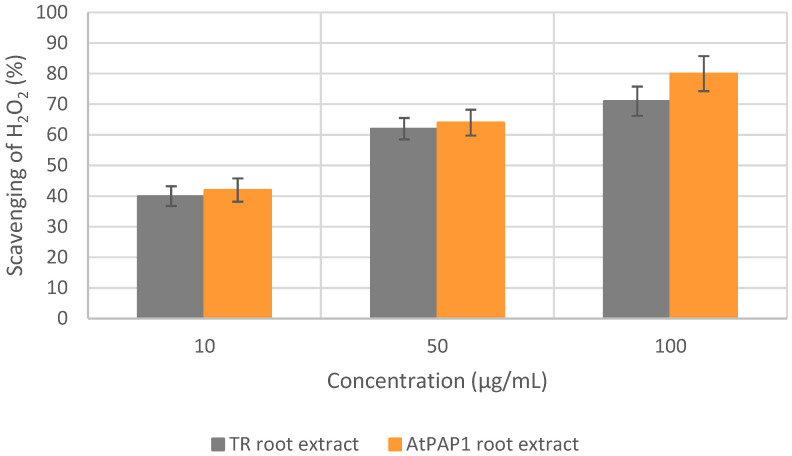
H_2_O_2_ scavenging activity of TR and AtPAP1 root extracts of *L. sibiricus* L.

In the H_2_O_2_ scavenging assay, the IC_50_ values of the TR and AtPAP1 *L. sibiricus* L. root extracts were determined to be 37.5 and 29.3 µg/mL, respectively. As a reference, the standard ascorbic acid demonstrated an IC_50_ value of 16.8 µg/mL. The dose-dependent scavenging effect of the *L. sibiricus* L. extracts on hydrogen peroxide is illustrated in Figure 2.

### 2.4. Nitric Oxide Scavenging Activity

**Figure 3:**
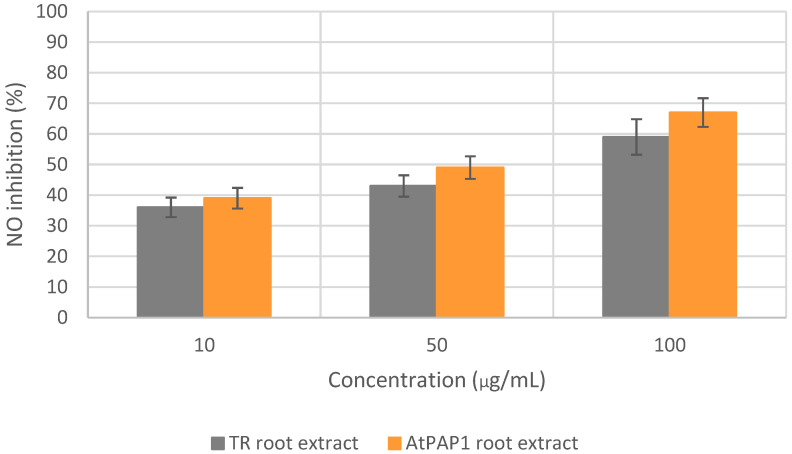
NO scavenging activity of TR and AtPAP1 root extracts of *L. sibiricus* L.

In the NO scavenging assay, the IC_50_ values of the TR and AtPAP1 *L. sibiricus* L. root extracts were determined to be 68.8 and 48.0 µg/mL, respectively. As a reference, the standard ascorbic acid demonstrated an IC_50_ value of 18.7 µg/mL. The dose-dependent scavenging effect of the *L. sibiricus* L. extracts on nitric oxide is illustrated in Figure 3.

### 2.5. Computational Studies

#### 2.5.1. Bond Dissociation Enthalpy Values of the -OH Bonds

The antioxidant activity of molecules identified in the TR and AtPAP1 *L. sibiricus* L. root extracts was determined using density functional theory calculations (DFTs). Their mechanism of action is mainly based on the radical scavenging process, whose strength can be estimated by BDE. The lowest value of BDE corresponds to lower stability of the bond and thus infers stronger antioxidant properties. In our study, the H atom was removed from the various phenolic groups presented in Figure 1. The BDE (OH) values for radicals are summarized in Table 3.

#### 2.5.2. HOMO LUMO

The antioxidant activities can be also characterized by means of the frontier molecular orbitals: Highest Occupied Molecular Orbital (HOMO) and Lowest Unoccupied Molecular Orbital (LUMO). However, in the case of radicals, Singly Occupied Molecular Orbital (SOMO) should be considered rather than HOMO. However, there is some dispute in the literature, as some studies treat them equivalently [21] while others do not [22,23,24]. Our results show that the HOMO orbital of each radical is energetically lower than SOMO. Thus, in this work, we analyze the HOMO-LUMO gap, focusing only on the α-spin and treating it as an indicator of antioxidant activity [25]. The values of HOMO, LUMO and their gap are listed in Table 4.

### 2.6. Anti-Inflammatory Activity

The anti-inflammatory effects of TR and AtPAP1 *L. sibiricus* L. root extracts were assessed based on their inhibitory activities against COX-2 and 5-LOX enzymes. In the initial screening, both extracts exhibited in vitro inhibitory effects on both enzymes (Table 5). 

### 2.7. Molecular Docking

**Figure 4:**
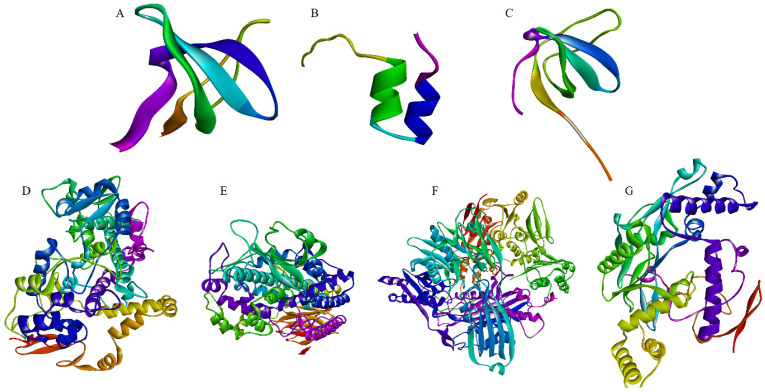
The target proteins: NOX p40phox (**A**), NOX p47phox (**B**), NOX p67phox (**C**), COX-2 (**D**), 5-LOX (**E**) iNOS (**F**) and XO (**G**) (Protein DataBank, www.rcsb.org, accessed on 20 May 2023).

The bioactive compounds identified in the TR and AtPAP1 *L. sibiricus* L. root extracts were subjected to molecular docking with NOX, COX-2, 5-LOX, iNOS and XO proteins (Figure 4). Phenol was used as a negative control. Typically, the binding pose that exhibits the lowest binding energy (indicating the highest binding affinity) is generally regarded as the optimal pose. The specific binding pose of a compound dictates its interaction with particular amino acids.

**Figure 5:**
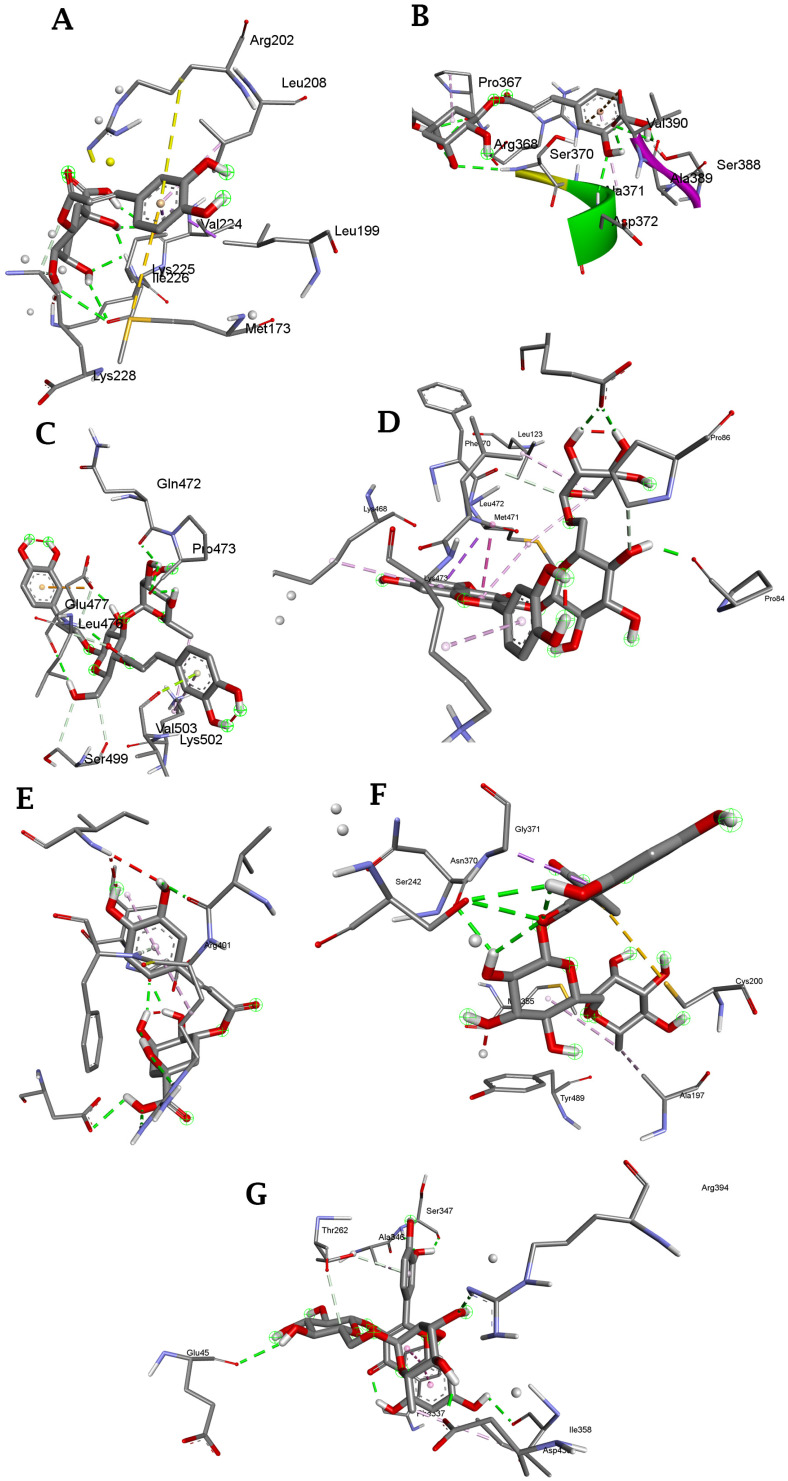
Compounds identified in *L. sibiricus* L. root extracts dock into targeted proteins with the lowest energy: (**A**): NOX p40phox with chlorogenic acid; (**B**): NOX p47phox with chlorogenic acid; (**C**): NOX p67phox with verbascoside; (**D**): COX-2 with rutin; (**E**): 5-LOX with chlorogenic acid; (**F**): iNOS with rutin; (**G**): XO with rutin.

The binding analysis between NOX, COX-2, 5-LOX, iNOS and XO proteins and ligands revealed that the binding pattern varied depending on the nature of the ligands. The docking results are represented in the form of minimum binding energy values, hydrogen bonding and other nonbonding interactions (Table 6). The ligands docked into target proteins are presented in Figure 5.

The evaluated binding energies ranged from −4.65 to −17.91 kcal/mol. The lowest binding energy was exhibited by chlorogenic acid docked into NOX p40phox (−7.87 kcal/mol), NOX p47phox (−7.48 kcal/mol) and 5-LOX (−13.36 kcal/mol), verbascoside docked into NOX p67phox (−10.97 kcal/mol) as well as rutin docked into COX-2 (−13.83 kcal/mol), iNOS (−17.91 kcal/mol) and XO (−12.32 kcal/mol). NOX, COX-2, 5-LOX, iNOS and XO play significant roles as factors involved in the inflammatory cascade and are specific targets for the molecular docking of various compounds, including those of plant origin.

## 3. Discussion

Plants have long been recognized as an important source of bioactive compounds, particularly antioxidants. These natural antioxidants derived from plants play a pivotal role in neutralizing free radicals and enhancing various biological functions. The secondary metabolites originating from plants are also of great interest due to their potential therapeutic properties. Previous studies have reported the identification of secondary metabolites from the roots of *L. sibiricus* L. [19]. Moreover, the *R. rhizogenes*-mediated transformation has been shown to enhance the synthesis of phenolics [26]. Additionally, the incorporation of the *Arabidopsis thaliana* PAP1 gene into the *L. sibiricus* L. genome via *R. rhizogenes*-mediated transformation, utilizing the control binary vector pCAMBIA 1305.1, offers an even greater enrichment of phenolics [4,27]. Recently, researchers have shown a profound interest in evaluating the antioxidant activity of these compounds using various available methods.

Phenolics are crucial plant molecules possessing redox properties that contribute to their antioxidant activity. The hydroxyl groups within these compounds play a significant role in facilitating free radical scavenging [7,28,29]. The Folin Ciocalteu colorimetric assay identified substantial amounts of phenolics in both the TR and AtPAP1 *L. sibiricus* L. root extracts, with the highest concentration observed in the AtPAP1 extract. Compared with the previous literature, Oliveira et al. reported a TPC of 60.1 mg of GAE/g in the ethanolic aerial parts extract of *L. sibiricus* L. [3] while Park determined a TPC of 0.59 g of chlorogenic acid/100 g in the aqueous extract of *L. sibiricus* L. leaves [30]. Barman et al. found TPC of 45.6 and 33.2 mg of GAE/g in the ethyl acetate and methanolic extracts of the whole *L. sibiricus* L. plant, respectively [31]. 

Flavonoids are an important group of phenolics recognized as effective antioxidants or free radical scavengers [32,33]. The aluminum chloride colorimetric assay revealed substantial amounts of flavonoids in both the TR and AtPAP1 *L. sibiricus* L. root extracts, with the highest concentration observed in the AtPAP1 extract. In the previous literature, Oliveira et al. reported a TFC of 15.4 mg catechin equivalent/g in the ethanolic extract of the aerial parts of *L. sibiricus* L. [3]. In studies of the whole *L. sibiricus* L. plant, Barman et al. determined a TFC of 68.3 and 42.2 mg of QUE/g in the ethyl acetate and methanolic extracts, respectively [31].

Hydrogen peroxide is recognized as a potent oxidizing agent capable of activating cellular signaling pathways to induce cellular proliferation [34] or differentiation [35]. Within biological systems, H_2_O_2_ is produced by various enzymes, including superoxide dismutase (SOD). SOD comprises a group of enzymes that catalyze the dismutation of superoxide radicals to molecular oxygen and hydrogen peroxide, providing cellular defense against ROS [36]. However, the aberrant accumulation of H_2_O_2_ can lead to oxidative stress due to its rapid decomposition and subsequent generation of the hydroxyl radical, which initiates damage to cellular components [37]. Therefore, significant attention has been paid to the regulation of H_2_O_2_ production by plant-derived antioxidants. This analysis showed that the TR and AtPAP1 *L. sibiricus* L. root extracts demonstrated strong H_2_O_2_ scavenging activity. Compared with the previous literature, Barman et al. determined IC_50_ values of 49.2 and 29.8 µg/mL or the ethyl acetate and methanolic extracts of the whole *L. sibiricus* L. plant, respectively [31]. Furthermore, nitric oxide plays a crucial role as a key regulator in various physiological functions such as cardiovascular homeostasis, metabolism, neurotransmission and immunity [38]. Within biological systems, NO is produced by enzymes called nitric oxide synthetases (NOSs), which convert the amino acid L-arginine into NO and another amino acid, L-citrulline. However, dysregulated NO signaling is associated with various pathophysiological abnormalities [39]. Therefore, the regulation of NO scavenging by plant compounds has significant attention in the field of biological research. This analysis showed that the TR and AtPAP1 *L. sibiricus* L. root extracts revealed strong NO scavenging activity in this assay. In previous studies, Oliveira et al. reported IC_50_ values of 266.6 µg/mL for the ethanolic aerial parts extract of *L. sibiricus* L. Barman et al. determined IC_50_ values of 29.2 and 36.6 for the ethyl acetate and methanolic extracts of the entire *L. sibiricus* L. plant, respectively [31]. Furthermore, the excessive production of both H_2_O_2_ and NO can induce inflammation, which is strongly associated with numerous disorders such as cancer, diabetes and cardiovascular diseases [40].

The DFT methods were used to assess the antioxidant activity of molecules identified in the TR and AtPAP1 *L. sibiricus* L. root extracts. Their mechanism of action is mainly based on the radical scavenging process, whose strength can be estimated by BDE. A low BDE value indicates lower bond stability and hence stronger antioxidant properties. Although the BDE values are of the same order of magnitude for all radicals, they are slightly lower for flavonoids, indicating that they are more potent antioxidants than phenolic acids. This can be reasoned by the fact that flavonoids contain one more phenolic group, and as is known, their number affects the antioxidant activity [41]. The hydrogen is most easily detached from the 3′ and 4′-OH sites of flavonoids, which is in line with the experimental observations. In the case of phenolic acids, hydrogen abstraction is only possible from the 3-OH and 4-OH sites; however, it is difficult to determine which one is more preferred as their BDE values are similar. An interesting situation occurs for the radicals of verbascoside because the three positions have almost the same BDE value: ~25 kcal/mol. Several theoretical studies describing antioxidant properties based on BDE are described in the literature. For instance, such studies have been performed for rutin [42]. The lowest BDE values were obtained for the 3′-OH of rutin, which contradicts our present findings. However, this position might be considered “equal”, as both belong to the same ring of flavonoids [43], which is indicated by many studies as the most important site for H-transfer. Other factors also have a significant impact on the final BDE value, such as the basis set used in calculations or the influence of the internal hydrogen bonds. 

The antioxidant activities can also be characterized by means of the frontier molecular orbitals: HOMO and LUMO. The results of the HOMO-LUMO gap are relatively well correlated with the BDE values for such compounds as catechin, chlorogenic acid and caffeic acid, indicating that the same radicals are the most potent antioxidants. Some differences are noted for rutin, as according to the HOMO-LUMO gap, the most reactive site is 3′-OH, while BDE points to 4′-OH. However, both positions have very similar values of the HOMO-LUMO gap and, as mentioned earlier, belong to the same ring [43]. 

Several studies have shown a positive association between antioxidants and anti-inflammatory properties among plant-derived compounds [44,45]. The TR and AtPAP1 *L. sibiricus* L. root extracts exhibited potent antioxidant activities. Additionally, the COX-2 and 5-LOX inhibition results suggest that the TR and AtPAP1 *L. sibiricus* L. root extracts may possess anti-inflammatory properties. This potential could stem from the compounds present in the extracts, which may inhibit various inflammatory mediators. Therefore, anti-inflammatory properties of the identified compounds were subjected to further study based on molecular docking to determine the ligand-protein interaction profiles. Five proteins involved in inflammatory process were engaged: NOX, COX-2, 5-LOX, iNOS and XO. Phytocompounds were evaluated based on their free energy of binding. The free energy of binding is equal to the free energy of the protein–ligand complex minus the free energies of the protein and the ligand in their unbound states; the value is used to indicate the degree of spontaneity and strength of protein–ligand binding. Typically, the binding pose that exhibits the lowest binding energy (indicating the highest binding affinity) is generally regarded as the optimal pose. The obtained docking results indicate that chlorogenic acid may have a modulatory role into NOX (p40phox and p47phox) and 5-LOX activity, verbascoside into NOX (p67phox) activity and rutin into COX-2, iNOS and XO activity due to the features of the interactions with amino acid residues and allocation into the active site. Enzymes are one of the most important groups of active molecule targets, and the identification of possible ligand–enzyme interactions is of major importance in many active molecules’ discovery processes.

## 4. Materials and Methods

### 4.1. Preparation of L. sibiricus L. Plant Extracts

The transformed roots were obtained by the infection of in vitro shoots of *L. sibiricus* L. with *R. rhizogenes* strain A4. The inoculation process for TR root cultures has been previously documented [19]. Transgenic hairy cultures with overexpression of AtPAP1 transcriptional factor were established based on previously described methods [20]. Lyophilized and powdered TR and AtPAP1 roots were subjected to extraction. The roots were immersed in 80% (*v*/*v*) aqueous methanol at 35 °C using an ultrasonic bath for 15 min, followed by two additional extractions with the same solvent for 15 min each. The resulting extracts were then filtered, combined and subjected to evaporation under reduced pressure. Subsequently, the extracts were lyophilized to dryness and stored in darkness until further use.

### 4.2. Quantification of Total Phenolic Content of L. sibiricus L. Root Extracts

The total phenolic content of TR and AtPAP1 root extracts was determined using the Folin–Ciocalteau colorimetric assay [46] with slight modifications using gallic acid as a standard to prepare a calibration curve. Briefly, 0.5 mL of extract (400 µg/mL) solution was mixed with 2.5 mL of 10% Folin–Ciocalteu reagent and 2 mL of 7.5% of sodium carbonate solution. The reaction mixture was allowed to incubate for a duration of 1 hour, after which the absorbance of the mixture was determined at 760 nm using a Shimadzu UV-1700 UV/VIS spectrophotometer (Tokyo, Japan), with a methanol blank solution serving as the reference. Total phenolic content was expressed as mg of gallic acid equivalent (GAE)/g of dried plant mass.

### 4.3. Quantification of Total Flavonoid Content of L. sibiricus L. Root Extracts

The total flavonoid content of TR and AtPAP1 root extracts was determined using the aluminum chloride colorimetric assay [47] with a slight modification using quercetin as a standard to prepare a calibration curve. Briefly, 2 mL of a methanolic solution containing 2% AlCl_3_ was combined with an equal volume of extract (100 μg/mL), shaken well and held for 10 min. The reaction mixture was allowed to incubate for a duration of 10 min, after which the absorbance of the mixture was determined at 415 nm using a Shimadzu UV-1700 UV/VIS spectrophotometer (Tokyo, Japan), with a methanol blank solution serving as the reference. Total flavonoid content was expressed as mg of quercetin equivalent (QUE)/g of dried plant mass.

### 4.4. Estimation of Hydrogen Peroxide Scavenging Activity of L. sibiricus L. Root Extracts

The hydrogen peroxide scavenging activity of TR and AtPAP1 root extracts was determined using the H_2_O_2_ assay [48] with slight modifications. Briefly, 2 mL of extract solution (10, 50 and 100 µg/mL) in methanol was added to 4.0 mL of H_2_O_2_ (20 mM) solution in phosphate buffer (pH 7.4). The reaction mixture was allowed to incubate for a duration of 10 min, after which the absorbance of the mixture was determined at 230 nm using a Shimadzu UV-1700 UV/VIS spectrophotometer (Tokyo, Japan), with a phosphate buffer blank solution serving as the reference. Ascorbic acid was used as a positive control. The percentage scavenging of H_2_O_2_ was calculated using Equation (1):

% scavenging of H_2_O_2_ = [(A0 − A1)/A0] × 100 (1)

where A0 was absorbance of the control and A1 was absorbance of the test extracts.

### 4.5. Estimation of Nitric Oxide Scavenging Activity of L. sibiricus L. Root Extracts

The nitric oxide scavenging activity of TR and AtPAP1 root extracts was determined using the Griess reagent [49] with slight modifications. In an aqueous solution, the generation of nitric oxide was achieved through the reaction of sodium nitroprusside with oxygen under physiological pH conditions. The resulting nitrite ions were quantified using Griess reagent, composed of 1% sulphanilamide, 2% phosphoric acid and 0.1% naphthylethylenediamine dihydrochloride. Briefly, 1 mL of extract solution (10, 50 and 100 µg/mL) was mixed with 0.5 mL of 10 mM sodium nitroprusside in phosphate buffer (pH 7.4). After 3 hours, the solution was mixed with 1.5 mL of Griess reagent. The reaction mixture was allowed to incubate for a duration of 10 min, after which the absorbance of the mixture was determined at 546 nm using a Shimadzu UV-1700 UV/VIS spectrophotometer (Tokyo, Japan), with a phosphate buffer solution serving as the reference. Ascorbic acid was used as positive control. The nitric oxide radicals scavenging ability was determined using the following Equation (2):

% Inhibition = (A0 − A1)/A0 × 100(2)

where A0 was the absorbance of the control and A1 was the absorbance of the test extracts.

### 4.6. Computational Details

In order to evaluate the antioxidant activities of molecules identified in the TR and AtPAP1 *L. sibiricus* L. root extracts, the HAT mechanism was taken into account. In this one-step reaction, a hydrogen atom is transferred from the phenolic group to a radical [50,51], as shown in Equation (3):

R^•^ + PhOH→RH + PhO^•^ (3)

where R^•^ and PhO^•^ are the free and phenolic radicals, respectively, while PhOH is a molecule that contains a phenolic group. 

In HAT, the strength of the radical scavenging process is estimated by calculating bond dissociation enthalpy (BDE), expressed as a difference in heat of formation between the molecule and its corresponding radical [52]. The BDE values were obtained based on density functional theory calculations (DFTs). A number of theoretical studies indicate that BDE is strongly dependent on the method used, especially the basis set [53,54,55,56,57]. In a work [43], the most accurate values of BDE were obtained from the two methods: B3LYP/6-311++G(2d,2p) and B3LYP/6-311++G(d,p), which contain the hybrid functional B3LYP [58] and People’s triple-zeta basis set. The present study considered various basis sets containing both diffusion and multiple polarization functions, however, such calculations failed due to severe numerical problems. Therefore, geometry optimization of all structures in their closed-shell singlet ground states was performed at B3LYP-D3/6-311G(d,p) level of theory in a gas phase. D3 denotes the empirical Grimme dispersion corrections (D3) [59], which were included in the calculations due to their importance in the radical study [60]. Radicals represent an open-shell system, so an unrestricted formalism was used in the calculations. During optimizations, the ultrafine integration grids were taken into account to increase the accuracy of the numerical integration. The same theory level was applied in the harmonic vibrational frequency calculations performed at 298 K to obtain enthalpy (H) and verify whether the optimized structures correspond to true minima on the potential energy surface. All calculations were conducted in the Gaussian09 program (Revision D.01) [61].

The enthalpy of formation (*H_f_*) was calculated using method based on the atomization energies, expressed as follows, Equation (4):

(4) $${\Delta H}_{f,298}^{˚}\left(C_{a}H_{b}O_{c}\right)=\left[a{\Delta H}_{f,298}^{˚}\left(C\right)+b{\Delta H}_{f,298}^{˚}\left(H\right)+{c\Delta H}_{f,298}^{˚}\left(O\right)\right]-\left[aH^{298}\left(C\right)+bH^{298}\left(H\right)+aH^{298}\left(O\right)\right]+H^{298}\left(C_{a}H_{b}O_{c}\right)$$

where *a*, *b* and *c* are the numbers of atoms in the molecule $C_{a}H_{b}O_{c}$, ${\Delta H}_{f,298}^{˚}$ is the measured heat of formation taken from JANAF tables [62] and $H^{298}$ is the enthalpy obtained from the B3LYP-D3/6-311G(d,p) calculations.

### 4.7. COX-2 and 5-LOX Inhibition Assays

The TR and AtPAP1 *L. sibiricus* L. root extracts were assessed for COX-2 inhibitory activity using a COX Inhibitor Screening Assay Kit (Cayman Chemical, Ann Arbor, MI, USA) and LOX inhibitory activity using a LOX Inhibitor Screening Assay Kit (Cayman Chemical, USA) as per the manufacturer’s protocol. The extracts were examined at concentrations of 100 µg/mL. Indomethacin (100 µM) and nordihydroguaiaretic acid (100 µM) were employed as positive controls for COX-2 and LOX, respectively. Absorbance at 415 nm and 490 nm was measured for COX-2 and LOX, respectively, using a plate reader (PerkinElmer, Waltham, MA, USA).

### 4.8. Molecular Docking Studies

Molecular docking serves the purpose of identifying the optimal arrangement of a ligand and a protein. Eight bioactive compounds identified from *L. sibiricus* L. hairy root extract with and without ATPAP1 were selected for molecular docking analysis: catechin, rutin, caffeic acid, coumaric acid, chlorogenic acid, ferulic acid and verbascoside. The chemical structures of the chosen bioactive compounds were taken from the PubChem compound database at NCBI (http://pubchem.ncbi.nlm.nih.gov/, accessed on 5 May 2023). Five proteins, NOX, COX-2, 5-LOX iNOS and XO were selected for their interactions with the bioactive compounds. All enzymes play a significant role in the modulation of inflammation. The 3D X-ray crystal structure of the proteins were retrieved from the Protein Data Bank (PDB ID: 1W6X–NOX p40phox; 1K4U–NOX p47phox chain s; 1K4U–NOX p67phox chain p; 5F1A–COX-2; 3O8Y–5-LOX; 4NOS–iNOS; 2CKJ–XO). All the structures were cleaned and optimized by removing ligand and other hetero-atoms using BIOVIA Discovery Studio Visualizer (version 21.1.0.20298) [63]. The active site residues was carried out using Computed Atlas of Surface Topography of proteins (CASTp) (http://stsfw.bioengr.uic.edu/castp/calculation.php, accessed on 20 May 2023) [64]. Phenol was used as a negative control. Then, the bioactive compounds were docked on the active site of target proteins using AutoDock software (version 1.5.7) [65]. Missing residues and atoms were added. Polar hydrogen and Gasteiger charges were subsequently made available for all proteins. According to the co-crystalized ligands of all proteins, the docking search grid boxes were determined to active site residues with a 60 Å3 total size. The grid box’s coordinates were set to be: x = −6.467, y = −0.999, z = 4.816 for NOX p40phox; x = 5.782, y = 6.699, z = 5.898 for NOX p47phox; x = −0.799; y = −2.544; z = −8.59 for NOX p67phox; x = 38.061, y = 15.122, z = 234.817 for COX-2; x = 14.729, y = 15.599, z = −17.400 for 5-LOX; x = −0.477, y = 95.913, z = 16.463 for iNOS; x = 93.693, y = 104.445, z = 133.621 for XO. The docking procedure was conducted following a conventional protocol utilizing the Lamarckian Genetic Algorithm [13]. One hundred independent docking runs were performed for each ligand. The various conformations for ligands in the docking procedure were generated and the final energy refinement of the ligand pose was performed. The docking score for the most favorable pose into the target proteins was computed for all the bioactive compounds under investigation. The visualization of interactions within ligand–receptor docked complex was performed by BIOVIA Discovery Studio Visualizer [63].

### 4.9. Statistical Analysis

All the experiments were carried out in triplicate, and the findings were reported as mean ± SD. The IC_50_ values were measured using the Microsoft Excel program. A *p*-value < 0.05 was considered significant.

## 5. Conclusions

The experimental results reported that *L. sibiricus* L. transgenic roots extracts transformed by *R. rhizogenes*, with and without the AtPAP1 transcriptional factor, are a possible source of molecules with antioxidant properties and displaying scavenging activity towards hydrogen peroxide and nitric oxide. Antioxidants are important molecules that can protect tissues against damage. Moreover, evidence shows that the root extracts could inhibit both COX and 5-LOX activity. Molecular docking studies of the identified compounds found that chlorogenic acid, verbascoside and rutin demonstrates promising binding affinity towards the NOX, COX-2, 5-LOX, iNOS and XO proteins and may act as potential inhibitors. They are important enzymes related to inflammatory processes. These results suggest that *L. sibiricus* L. root extracts may also have significant anti-inflammatory properties. However, further in vitro and in vivo studies of the inflammatory aspects of the extract are essential to determine its potential applications.

## Figures and Tables

**Table 1:** The contents of secondary compounds identified in the TR and AtPAP1 *L. sibiricus* L. root extracts presented as µg/g dry weight (DW).

Compounds	TR Extract (µg/g DW)	AtPAP1 Extract (µg/g DW)
chlorogenic acid	4104 ± 8.7	19,392 ± 110.1
caffeic acid	4176 ± 9.0	11,380 ± 136.6
*p*-coumaric acid	30 ± 0.1	52 ± 1.1
ferulic acid	660 ± 27.1	1172 ± 36.3
4-hydroxybenzoic acid	48 ± 1.2	53 ± 1.5
D-(+)-catechin	120 ± 2.3	185 ± 1.8
rutin	870 ± 13.2	890 ± 11.4
verbascoside	1473 ± 17.7	-

**Table 2:** Total phenolic (TPC) and flavonoid (TFC) content in the TR and AtPAP1 *L. sibiricus* L. root extracts.

Extract	TPC (mg GAE/g)	TFC (mg QUE/g)
TR root extract	57.4 ± 0.15	18.2 ± 0.44
AtPAP1 root extract	85.3 ± 0.35	25.6 ± 0.42

**Table 3:** Calculated values of the bond dissociation enthalpy (BDE) for different radicals formed after H removal from the different -OH positions.

Radicals	BDE (kcal/mol)
catechin (3′-OH)	30.53
catechin (4′-OH)	22.93
catechin (5-OH)	29.12
catechin (7-OH)	30.65
chlorogenic acid (3′-OH)	22.98
chlorogenic acid (4′-OH)	29.28
ferulic acid (4-OH)	22.85
4-hydrobenzoic acid	33.43
caffeic acid (3-OH)	28.26
caffeic acid (4-OH)	25.66
*p*-coumaric acid (4-OH)	29.50
rutin (3′-OH)	27.42
rutin (4′-OH)	22.62
rutin (5-OH)	33.68
rutin (7-OH)	34.35
verbascoside (3″-OH)	25.93
verbascoside (4″-OH)	25.03
verbascoside (3‴-OH)	28.35
verbascoside (4‴-OH)	25.00

**Table 4:** The values of HOMO, LUMO and HOMO-LUMO gap calculated at B3LYP-D3/6-311G(d,p) theory level.

Radicals	HOMO (Hartree)	LUMO (Hartree)	HOMO-LUMO Gap (eV)
catechin (3′-OH)	−0.23	−0.03	5.34
catechin (4′-OH)	−0.22	−0.03	5.21
catechin (5-OH)	−0.23	−0.02	5.79
catechin (7-OH)	−0.23	−0.02	5.65
chlorogenic acid (3′-OH)	−0.27	−0.09	4.77
chlorogenic acid (4′-OH)	−0.27	−0.08	5.03
ferulic acid (4-OH)	−0.27	−0.08	5.12
4-hydrobenzoic acid	−0.28	−0.06	6.03
caffeic acid (3-OH)	−0.27	−0.09	4.85
caffeic acid (4-OH)	−0.27	−0.08	5.16
*p*-coumaric acid (4-OH)	−0.27	−0.09	5.09
rutin (3′-OH)	−0.24	−0.07	4.38
rutin (4′-OH)	−0.24	−0.08	4.58
rutin (5-OH)	−0.24	−0.07	4.59
rutin (7-OH)	−0.24	−0.08	4.47
verbascoside (3″-OH)	−0.23	−0.08	4.01
verbascoside (4″-OH)	−0.23	−0.09	4.01
verbascoside (3‴-OH)	−0.24	−0.10	3.78
verbascoside (4‴-OH)	−0.23	−0.09	3.79

**Table 5:** Anti-cyclooxygenase and anti-lipoxygenase activity of TR and AtPAP1 *L. sibiricus* L. root extracts.

Sample	COX-2 Inhibition (%)	5-LOX Inhibition (%)
TR root extract	61.7 ± 7.2	54.3 ± 3.1
AtPAP1 root extract	65.4 ± 5.3	52.6 ± 4.2
Indomethacin	81.3 ± 3.9	-
Nordihydroguaiaretic acid	-	98.0 ± 3.6

**Table 6:** The binding energies, hydrogen bonds and nonbonding interactions obtained from the molecular docking performed between the investigated compounds and the selected proteins.

Compound Name	Binding Free Energy (kcal/mol)	Hydrogen Bonding	Other Nonbonding Interaction
**NOX p40phox**			
4-hydroxybenzoic acid	−4.65	VAL196, VAL21232	ARG174
caffeic acid	−5.39	ASP206, VAL224	LEU221, SER222
catechin	−7.11	MET173, LEU199	ARG202, LEU208, ILE226, LYS228
chlorogenic acid	−7.87	VAL224, ILE226	MET173, LEU199, ARG202, LEU208, LYS225, LYS228
ferulic acid	−5.55	LEU199	MET173, ARG202, LEU208, ILE226, LYS228
*p*-coumaric acid	−5.10	LEU199, ARG202	LEU208
rutin	−6.59	MET173, LEU200, ARG202	LEU199, LEU208, ILE226
verbascoside	−5.77	ILE226	LEU221, VAL224, LYS225, LYS228
phenol	−4.04	-	-
**NOX p47phox**			
4-hydroxybenzoic acid	−4.84	ARG368, PRO369, ALA371, LYS385	LEU386
caffeic acid	−5.75	ARG368, ASP372, VAL390, ALA389	ALA371
catechin	−7.09	ARG368, PRO369, LYS385, SER388	ALA371, ILE374, LEU386
chlorogenic acid	−7.48	PRO367, SER370, ASP372, SER388, ALA389, VAL390	ARG368, ALA371
ferulic acid	−5.41	ALA371, SER388, VAL390	ARG368
*p*-coumaric acid	−4.95	PRO367, SER388, VAL390	SER370
rutin	−6.31	-	ARG368, PRO369, ALA371, ILE374, LEU386, CYS378
verbascoside	−6.85	LEU373, LYS385	PRO366, PRO369, ARG377, ALA371, LEU386
phenol	−3.69	-	-
**NOX p67phox**			
4-hydroxybenzoic acid	−6.16	PRO473, ASP475, SER499, GLY504	LEU476, GLU477, GLU498, LYS502
caffeic acid	−7.04	SER499, GLN472, PRO473, ASP475	LEU476, LYS502
catechin	−9.13	ALA 470, GLN472, PRO473, GLU477, SER499	LEU476, LYS502
chlorogenic acid	−10.29	PRO473, GLU477, GLN479, LYS502	LEU476, PHE478, GLY504
ferulic acid	−6.95	GLN472, PRO473, SER499	LEU476, GLU477, LYS502
*p*-coumaric acid	−6.32	GLN472, PRO473, SER499	LEU476, LYS502
rutin	−10.74	THR471, GLN472, PRO473, ASP475, GLU477, ASP482, SER499	LEU476, LYS500, LYS502
verbascoside	−10.97	GLN472, PRO473, GLU477	LEU476, SER499, LYS502, VAL503
phenol	−4.70	-	-
**COX-2**			
4-hydroxybenzoic acid	−7.29	VAL228, ASN375, SER530, GLY533	PHE209, GLY227, LEU534
caffeic acid	−9.08	TYR385	PHE209, GLY227, ASN375, LEU534
catechin	−12.23	HIS39, ASN43, GLY45, CYS47, GLN461, GLU465, LYS468	ARG44, LEU152
chlorogenic acid	−11.79	CYS41, GLY45, LYS468	ARG44, PRO153
ferulic acid	−9.07	ASN375, TYR385	GLY227, ILE377, PHE381, PHE529, LEU534
*p*-coumaric acid	−8.30	ASN375, ARG376	PHE209, ALA378, LEU534, GLY533
rutin	−13.83	PRO84, GLU524	PRO86, LEU123, LYS468, PHE470, MET471, LEU472, LYS473
verbascoside	−10.14	THR60	ARG44, ARG61, GLY63, MET471, LEU472
phenol	−5.35	-	-
**5-LOX**			
4-hydroxybenzoic acid	−7.73	ASP166, ILE167, ILE404	PHE402
caffeic acid	−9.37	TRP102, ILE167, GLN168, VAL400, ILE404	PHE402
catechin	−12.63	TRP102, ILE167, ASP170, TYR383, ILE404	GLN15, TYR81, ARG401, PHE402, GLU622
chlorogenic acid	−13.36	ASP166, ASP170, VAL400, ARG401	ILE167, PHE402, ILE404
ferulic acid	−9.25	GLN15, ASP170, TTYR383	ILE167, VAL400, ARG401, PHE402, ILE404
*p*-coumaric acid	−8.33	TRP102, GLN168, VAL400	ILE167, PHE402
rutin	−12.30	SER14, ASP170, ASN613, GLU622	GLY13, GLN15
verbascoside	−11.81	GLN15, TYR81, TYR100, GLU614, MET619, GLU622	SER14, LYS83, TRP102, ASN613, LEU615, PRO621
phenol	−5.46	-	-
**iNOS**			
4-hydroxybenzoic acid	−6.90	LEU125	ALA197, PRO198, ARG199, MET355, TYR491
caffeic acid	−7.87	ASN370	TRP194, LEU209, PHE369
catechin	−11.34	TYR489	TRP194, ALA197, CYS200, PHE488, MET355, PHE369
chlorogenic acid	−13.52	PRO350, VAL352, TRP372	TRP194, CYS200, LEU209, PHE369, TYR489
ferulic acid	−8.56	TYR489, PRO350	THR190, TRP194, ALA197, LEU209, SER242, ILE244, ALA351, VAL352, ASN370, GLY371, TYR489,
*p*-coumaric acid	−7.50	TYR489, PRO350, VAL352	TRP194, LEU209
rutin	−17.91	SER242, ASP370	ALA197, CYS200, MET355, GLY371, TYR489
verbascoside	−15.06	TRP463, ASN370, TRP372, GLU377	TRP194, PHE369, MET374
phenol	−5.24	-	-
**XO**			
4-hydroxybenzoic acid	−7.40	ILE264, SER347	THR262, ALA346, GLY350, ASN351
caffeic acid	−8.53	LEU257, SER347	VAL258, VAL259, ILE264
catechin	−10.91	LEU257, SER347, GLU263, ILE264, ASN351	ALA346, GLY350, VAL258, THR262
chlorogenic acid	−12.05	LEU257, VAL259, SER347, ASN351, THR354	ALA346, GLY350
ferulic acid	−8.90	LEY257, SER347	VAL258, VAL259
*p*-coumaric acid	−7.52	LEU257, SER347	VAL258, VAL259
rutin	−12.32	GLU45, SER347, ILE358, ARG394, ASP430	PHE337, ALA346
verbascoside	−12.15	GLU45, THR262, ALA338, ASN351	GLY47, ILE266, VAL342, ALA346, SER347, GLY350, SER359
phenol	−4.83	-	-

## Data Availability

Not applicable.
